# The Royal Commission on Tuberculosis

**Published:** 1904-06-04

**Authors:** 


### THE ROYAL COMMISSION ON TUBERCULOSIS.

The interim report which has just been issued by the Royal Commission appointed in 1901 to inquire into the relations of human and animal tuberculosis, must be regarded as a most important document, setting forth as it does authoritatively certain facts relative to the transmissibility of human tuberculosis to animals, which must serve in the future to guide both the medical profession and, possibly, the Legislature as to the correct attitude to be adopted towards this subject when any steps for the prevention of tuberculosis are under consideration. It will be remembered that the Commission was appointed to inquire (1) Whether tuberculosis in animals and man is one and the same disease; (2) whether animals and man can be reciprocally infected with the disease; (3) under what conditions, if at all, the transmission of the disease from animals to man takes place, and what are the circumstances favourable or unfavourable to such transmission. This reference is, of course, directed mainly to settle the important question raised by Professor Koch whether the bacillus which gives rise to bovine tuberculosis is, or is not, specifically distinct from that which causes the disease in man. The Commissioners, namely Sir Michael Foster, M.P. (chairman), Professor G. Sims Woodhead, Professor Sidney Martin, Professor McFadyean, and Professor R. W. Boyce, after due consideration, decided not to begin the inquiry by collecting the opinion of others, but to attack the problem by conducting experimental investigations of their own, and to defer second-hand evidence till later on—an order of procedure which at once commends itself to the scientific mind. The first line of inquiry, which is the sole subject of the interim report, was to determine what effects are produced by introducing into cattle, either through the alimentary canal as food, or directly into the tissues by subcutaneous or other injection, tuberculous material obtained from various cases of tuberculous disease in human beings—and how far these effects resemble or differ from the effects produced by introducing into the bovine animal, under conditions as similar as possible, tuberculous material of bovine origin—*i.e.* material containing living tubercle bacilli obtained from cases of tuberculous disease in the cow, calf, or ox. For the purpose of these experiments more than two hundred bovine animals were made use of, and over twenty different "strains" of tuberculous material of human origin—that is to say, of material taken from more than twenty cases of tuberculous disease in human beings, including sputum from phthisical patients and the diseased parts of the lungs in pulmonary tuberculosis, mesenteric glands in primary abdominal tuberculosis, tuberculous bronchial and cervical glands, and tuberculous joints. The effects produced by these were compared with the effects produced by several different strains of tuberculous material of bovine origin.

The results are instructive. Not one of the strains failed to produce tuberculosis in animals subjected to inoculation, while in the case of seven of the strains, the introduction of the human tuberculous material gave rise at once to acute tuberculosis, with the development of widespread disease in various organs of the body, such as the lungs, spleen, liver, lymphatic glands, etc. In some instances the disease was of remarkable severity.

The Commission state that they have carefully compared the disease thus set up in the bovine animal by material of human origin with that set up in cattle by material of bovine origin, and so far have found the one, both in its general features and its histological details, to be identical with the other; and they have been unable to discover any character by which the one could be distinguished from the other. From these results it is clear that human tuberculosis is capable of causing disease in animals which is indistinguishable from bovine tuberculosis; and even if absolute proof is wanting that the latter disease can be ingrafted upon man, yet in view of the above facts it seems so probable that it may be, that until convincing proof be forthcoming to the contrary, it must be assumed that bovine tuberculosis is a source of danger, through possibility of infection, to the human being.

